# Gradient shadow pattern reveals refractive index of liquid

**DOI:** 10.1038/srep28191

**Published:** 2016-06-15

**Authors:** Wonkyoung Kim, Dong Sung Kim

**Affiliations:** 1 Department of Mechanical Engineering, Pohang University of Science and Technology (POSTECH), 77 Cheongam-ro, Pohang 37673, Republic of Korea

## Abstract

We propose a simple method that uses a gradient shadow pattern (GSP) to measure the refractive index *n*_*L*_ of liquids. A light source generates a “dark-bright-dark” GSP when it is projected through through the back of a transparent, rectangular block with a cylindrical chamber that is filled with a liquid sample. We found that there is a linear relationship between *n*_*L*_ and the proportion of the bright region in a GSP, which provides the basic principle of the proposed method. A wide range 1.33 ≤ *n*_*L* _≤ 1.46 of liquids was measured in the single measurement setup with error <0.01. The proposed method is simple but robust to illuminating conditions, and does not require for any expensive or precise optical components, so we expect that it will be useful in many portable measurement systems that use *n*_*L*_ to estimate attributes of liquid samples.

Refractive index *n* is a fundamental physical properties of a substance. *n* determines the path of light when it crosses an interface between two media, so its measurement is essential when designing and fabricating optical instruments such as optical lenses^1^,^2^,^3^, prisms, mirrors, and optical fibers. In addition, because refractive index *n*_*L*_ of a liquid strongly depends on the amounts or kinds of solutes in it^4^, measurement of *n*_*L*_ provides important information in many fields (e.g., sugar content in foods, degeneration of industrial oils, and diagnoses of disease^5^,^6^,^7^,^8^,^9^).

To date, various existing methods measure *n* of a substance by evaluating refraction angle, critical angle, interference pattern, or dielectric constant^10^. Interferometers and ellipsometers provide comprehensive information of n, but are too expensive and complicated to use as a simple portable refractometer. More recently, microfluidic-11,12,13,14,15, diffractive-16, and holographic-based^17^ systems to measure *n* have been developed; they have several advantages such as low sample volume, but they entail complex configurations and require precise and expensive optical components. Therefore, these systems are also inappropriate for portable applications. The most widely-used portable refractometer applies triangular prisms. While passing through the interface between a solid triangular prism with known *n* and a liquid sample of which *n*_*L*_ is to be determined, a beam of light is deviated, i.e., refracted or reflected. The amount of deviation is then converted to *n*_*L*_ of the liquid sample by performing calculations that consider *n* of the prism and its dimensions. Despite its simple principle, a setup to estimate the deviation angle is complicated, because it is usually conducted by a rotating telescope, photodetector, or goniometer^18^. Also, due to the planar interface between the prism and liquid sample, incident angle *θ*_1_ at the interface does not change much, so the measurement range of *n*_*L*_ is limited. To measure a wide range of *n*_*L*_, multiple prisms having different *n* should be used. Another solution to use a specialized illumination system that can widen the variation of *θ*_1_. Both of these solutions are expensive and increase the complexity of the measurement.

This paper presents an alternative simple method to measure *n*_*L*_. It does not require any expensive optical instruments or special illumination systems. The proposed method uses a conventional light source and a transparent, rectangular solid block with a cylindrical chamber that is filled with a liquid sample of interest. One novel feature is the circular interface between the solid block and the liquid sample that cause a continuous change of *θ*_1_ from 0° to ±90°. Projection of the light source through the block and the sample generates a “dark-bright-dark” spatial gradient shadow pattern (GSP). From a simple analysis of a GSP, a wide range 1.33 ≤ *n*_*L*_ ≤ 1.46 was easily measured in a single measurement setup. The measurements agreed well with *n*_*L*_ values obtained using a commercial refractometer, and the measurement error was <0.01. The effects of illumination conditions on the measurement accuracy were negligible. The proposed method has applications in portable measurement systems that require *n*_*L*_ to estimate attributes of liquids.

## Results

**Figure 1:**
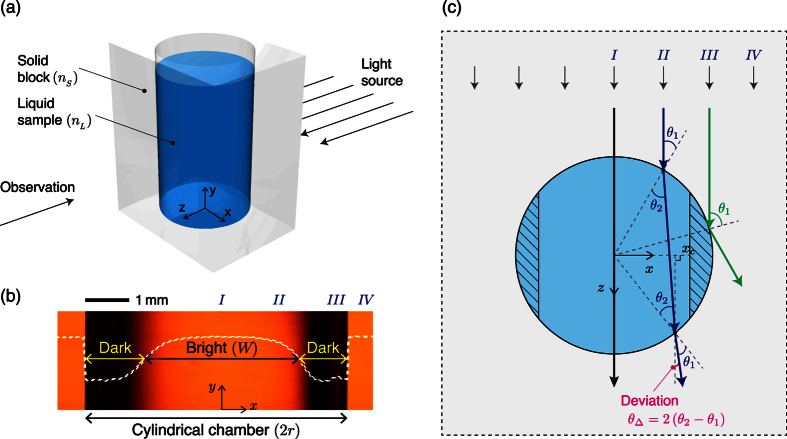
Proposed setup to measure refractive index *n*_*L*_ of liquids by exploiting a circular interface between a solid block and a liquid sample. (**a**) Schematic diagram of the setup for *n*_*L*_ measurement. A solid block has a cylindrical chamber which is filled with a liquid sample. When a light source is illuminated behind the block, a GSP appears. The schematic diagram was personally drawn by the authors. (**b**) Experimentally observed “dark-bright-dark” GSP. White broken line: brightness profile of the GSP; width (*W*) of the bright region is the basis for measurement of *n*_*L*_. (**c**) Schematic ray diagram of light passing through the measuring block. Light ray illuminated from around the center of the cylindrical chamber is refracted and spread out (dark blue line), whereas light rays that strike the side of the chamber are totally reflected (green line).

The measurement system (Fig. 1a) consists of a light source, an observation camera, and a transparent rectangular poly(methyl methacrylate) (PMMA) block (length 12 mm, width 10 mm, height 14 mm), into which a cylindrical chamber (radius *r* = 3 mm, depth 12 mm) has been drilled. The block has refractive index *n*_*S*_ = 1.49 at wavelength of 589.3 nm (sodium D line) at 20 °C. The cylindrical chamber is filled with a liquid sample of interest. The cylindrical shape of the sample chamber causes a continuous change in *θ*_1_ at the interface between the block and a liquid sample.

A light source projected through the block generated a “dark-bright-dark” GSP (Fig. 1b), in which the width of bright region is represented by *W*. The left and right sides of the cylindrical chamber were observed to be dark, whereas the center region is bright. The proportion *W*/(2*r*) of bright region in the GSP depends only on *n*_*L*_ and *n*_*S*_; the proposed method to measure *n*_*L*_ exploits this relationship. One of the novel features of the proposed method is adopting the curved interface between the solid block and the liquid sample which is the main factor in generating a GSP. When a triangular prism chamber is used instead of the cylindrical chamber, no GSP appears (Supplementary Fig. S1). To simply analyze the generation of the GSP, we considered a circle on a plane and traced paths of rays entering and leaving the circular interface (Fig. 1c). We did not consider the planar interfaces between the solid block and air, because this interface did not contribute to formation of the GSP.

Different light rays incident on the circular interface refract differently from the cylindrical chamber, so their deviation angles *θ*_Δ_, defined as the angle difference between the ray entering and leaving the circular interface (Fig. 1c), vary. As the light incidence position moves toward the sides of circular interface, *θ*_Δ_ increases sharply and thereby generates a nonlinear transformation of light intensity distribution. The light intensity is high (bright) at the center of the cylinder, but low (dim) at both sides. The paths of light rays can be classified into four regimes based on the position *x* of incident light on the circular interface (Fig. 1c).

### Regime I (central region)

When the light ray is illuminated at the central point (*x* = 0) of circular interface, *θ*_1_ = 0° and the light ray travels straight. Thus, the central region of GSP is bright.

### Regime II (diffuse region)

**Figure 2:**
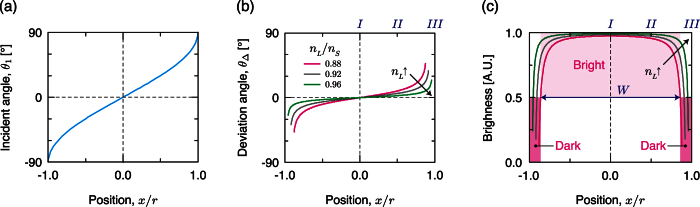
Generation of a “dark-bright-dark” GSP. (**a**) Continuous variation of incident angle *θ*_1_. Incident angle changes from 0° to ±90° depending on the position from the center of the cylindrical chamber (*x* = 0) to the sides (*x*/*r* = ±1). (**b**) The deviation angle *θ*_Δ_ = 2 (*θ*_2_ − *θ*_1_) of light ray because of different refraction from the cylindrical chamber. As the light incident position moves toward the sides of cylindrical chamber, *θ*_Δ_ increases sharply as a result of nonlinear transformation of incident light distribution. If as the refractive index *n*_*L*_ of the liquid sample approaches that of the solid block, *θ*_Δ_ decreases. (**c**) Brightness profiles in GSPs generated by various liquids having different *n*_*L*_. Because *θ*_1_ and *θ*_Δ_ are close to 0°, the brightness in the center region (*x* = 0) is high, but the brightness near the sides (*x* → *r*) is low due to the reduced amount of refracted light. The width (*W*) of the bright region increases as *n*_*L*_ increases.

When 0 < |*x*| < (*n*_*L*_/*n*_*S*_) the incident angle increases (Fig. 2a) as:


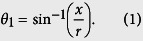


The incident ray is refracted twice (Fig. 1c) and the refraction angle


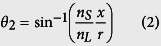


is obtained using Snell’s law. Deviation angle *θ*_Δ_ ≡ 2(*θ*_2_ − *θ*_1_) increases nonlinearly as path of the incident light ray approaches the sides of the chamber (Fig. 2b). Because of the spatial variation of *θ*_Δ_, the incident rays diverge while traveling the chamber, so the brightness of transmitted light decreases in this region.

### Regime III (dark region)

Beyond the critical points (*n*_*L*_/*n*_*S*_) ≤ |*x*| < *r*, *θ*_1_ exceeds the critical angle *θ*_*c*_ ≡ sin^−1^ (*n*_*L*_/*n*_*S*_), so the incident light is totally reflected (Fig. 1c). The shadow that corresponds to this region is completely dark.

### Regime IV (solid region)

Light rays in the region of *r* ≤ |*x*| pass through and miss the cylindrical chamber, so *θ*_Δ_ = 0. In this region, no shadow occurs (completely bright).

We analyzed the brightness profile in a GSP from the spatially-varying *θ*_Δ_ (Fig. 2c; details in Supplementary Information). In the central region (*x* = 0) the brightness is high because *θ*_1_ and *θ*_Δ_ are close to 0°. In contrast, the brightness at the sides (*x* → ±*r*) is low due to the deviated and spread light path. The trend of changes in *θ*_Δ_ and brightness profile in the GSP is strongly affected by the ratio *n*_*L*_/*n*_*S*_. When 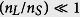, *θ*_*c*_ becomes small; as a result, the portion of dark region in the GSP expands. In contrast, as *n*_*L*_/*n*_*S*_ approaches 1, *θ*_*c*_ increases and *θ*_Δ_ decreases; this trend widens the bright region in the GSP widens (Fig. 2b,c). We do not consider *n*_*L*_/*n*_*S*_ > 1 in this study. The relationship between the proportion *W*/(2*r*) and *n*_*L*_/*n*_*S*_ was almost perfectly linear (Supplementary Fig. S2c).

**Figure 3:**
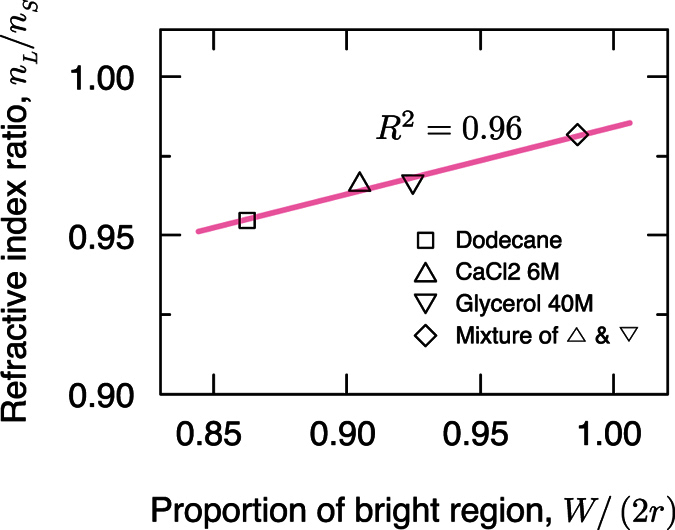
Proportion of bright region *W*/(2*r*) of GSPs with the change of the ratio *n*_*L*_/*n*_*S*_ of the refractive indices of liquid sample and of the solid block. Red line: linear fit, which is exploited to estimate the unknown refractive index of a liquid sample by analyzing corresponding GSP.

However, we ignored the refraction at the planar interfaces between the solid block and air, so a more realistic relationship between *W*/(2*r*) and *n*_*L*_/*n*_*S*_ should be obtained. In this regards, instead of deriving a theoretical relationship, we deduced the relationship by analyzing the GSPs obtained using liquids of known *n*_*L*_. The width of the bright region was evaluated as the full width at half maximum (FWHM) brightness in the GSPs. Measured *n*_*L*_/*n*_*S*_ ratio is found to have increased a linearly relationship with as *W*/(2*r*) increased proportion (*R*^2^ = 0.96, Fig. 3) as:


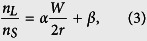


where *α* = 0.21 ± 0.03 and *β* = 0.77 ± 0.03.

**Figure 4:**
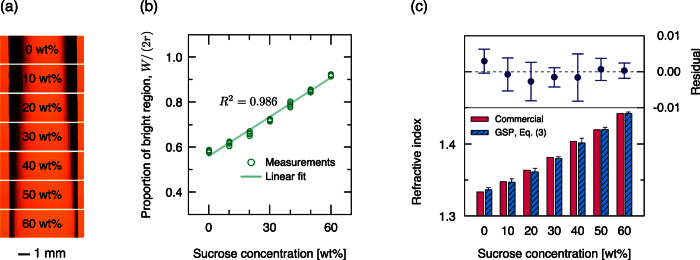
Measurement of refractive indices *n*_*L*_ of sucrose solutions with various concentrations from 0 to 60 wt%. (**a**) “Dark-bright-dark” GSPs of sucrose solutions. (**b**) Influence of the sucrose concentrations on the proportion of the bright region. The proportions increase linearly with the sucrose concentration, i.e., with *n*_*L*_. Each measurement was repeated eight times. (**c**) Comparison of *n*_*L*_ measured by a commercial refractometer (red solid bars) and by GSPs (blue patterned bars). Error bars: 95% confidential interval.

To evaluate the versatility of the proposed *n*_*L*_ measurement system, it was used to estimate *n*_*L*_ of sucrose solutions with various concentration and the results compared to *n*_*L*_ measured using the commercial refractometer. These sucrose solutions made different GSPs (Fig. 4a). Interestingly, the proportion of the bright region increased linearly with the sucrose concentration (*R*^2^ = 0.986, Fig. 4b). *n*_*L*_ measured using the proposed method agreed quantitatively with *n*_*L*_ obtained using the commercial refractometer (Fig. 4c) and reference *n*_*L*_ of sucrose solutions^19^; measurement error was <0.01, which can be regarded as the resolution of the present measurement setup. This result confirmed the feasibility of the proposed method. The results also indicate that the proposed method to measure *n*_*L*_ can be used quantify sugar content in foods, and perhaps to measure concentrations of other solutes in liquids.

Because the *n*_*L*_ measurement is based on the empirically-derived linear relationship of Eq. (3), its applicable range is of practical importance. The experimental demonstration already confirmed the validity of the linearity between *n*_*L*_/*n*_*S*_ and *W*/(2*r*) for 1.33 ≤ *n*_*L*_ ≤ 1.46. This measurement range is appropriate for liquids, because 1.33 is the lowest *n*_*L*_ value observed among common liquids^19^. We conducted the same measurement of air (*n* = 1.0), but the result did not agree with the linear relationship of Eq. (3) (data not shown). Because we have assumed that *n*_*L*_ < *n*_*S*_ when calculating the divergence of the incident light, the upper limit of measurable *n*_*L*_ is *n*_*S*_ (here, 1.49). In this case, all of the GSP is bright.

## Discussion

**Figure 5:**
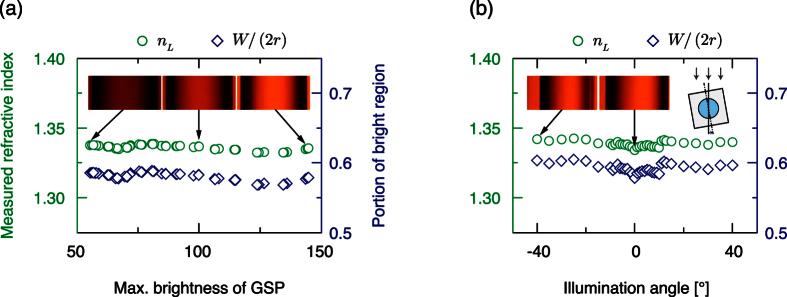
Effects of illumination (**a**) intensity and (**b**) angle of light source on estimates of *n*_*L*_. Proportion of bright region (navy diamond) and measured refractive index (green circle) are not affected by the illumination (**a**) intensity and (**b**) angle. Inset: images of GSP of DI water.

We further investigated the reliability of estimates of *n*_*L*_ obtained using the proposed method under different conditions of illuminance intensity, and direction of the light source. First, GSPs of DI water were collected at 16 illuminance intensities (Fig. 5a). There was no significant change in calculated *n*_*L*_ under different illuminance intensities. The striking thing is that the calculated *n*_*L*_ changed only 0.006, although *W* changed 0.12 mm. Because the change of *n*_*L*_ was lower than the resolution of the proposed measurement system, the effect of illuminance intensity can be neglected. Also, the linear relationship is still valid for various illuminance intensities.

Second, to examine the effect of illumination direction, we took GSPs of DI water obtained by rotating the solid block from −40° to 40° (Fig. 5b). As the angle of illumination direction increases, observed widths of the cylindrical hole and bright region decrease (Fig. S4). However, interestingly, the ratio of the two widths, or proportion of the bright region, was found to be almost constant regardless of the illumination angle (Fig. 5b). Maximum difference of calculated *n*_*L*_ was only 0.008, which is lower than the resolution of the proposed method. Therefore, we can conclude that estimate of *n*_*L*_ is not affected by the illumination angle from −40° to 40°. When the angle exceeds ±40°, a focused image of GSP cannot be obtained. Also, we found that the proposed method is little affected by the illumination color within the resolution range (±0.01) (Supplementary Fig. S3). However, in the absence of a color filter, estimate of *n*_*L*_ was slightly overestimated (+0.02). These results demonstrate that the proposed *n*_*L*_ measurement is not sensitive to illumination conditions, and imply that the proposed method is robust and can be used with natural light as a power-free portable device.

The resolution and measuring range of the proposed method are related to the quantity *αn*_*S*_/(2*r*) that relates *W* to *n*_*L*_ (Eq. (3)). As this quantity decreases, the resolution would improve but the measuring range would narrow, and vice versa. The GSP-based *n*_*L*_ measurement is simple but accurate, and is not sensitive to illumination conditions. It is also built using inexpensive components and does not requires an external power source or a specialized light source. Therefore, this device can be used in various power-free, portable, and even disposable devices. Also, it may be further modified for microfluidics applications such as detecting interfaces between two immiscible liquids or mixing interface between two miscible liquids that have different *n*_*L*_ flowing in a microchannel.

In conclusion, we proposed and demonstrated a simple method to measure the refractive index *n*_*L*_ of liquids. The method exploited a “dark-bright-dark” spatial GSP generated by a circular interface between solid and liquid. The circular interface caused a continuous change of incident angles from 0° to ±90°, and consequently generated a unique GSP. The proportion *W*/(2*r*) of bright region in the GSP was linearly related to *n*_*L*_/*n*_*S*_ and this relationship is exploited to estimate *n*_*L*_. The proposed method was accurate over the range 1.33 ≤ *n*_*L*_ ≤ 1.46, with error <0.01. Although the principle and structure of the proposed method is simple, it is accurate and robust to illumination conditions. Therefore, we expect that this device can be adapted to a variety of inexpensive (possibly disposable) portable instruments to measure *n*_*L*_.

## Method

Tests were conducted using liquid samples of various known *n*_*L*_ (Dodecane (Sigma-Aldrich, anhydrous 99+%, *n*_*L*_ = 1.42), 40 M glycerin (Daejung, 99+%) solution (*n*_*L*_ = 1.44), 6 M CaCl_2_ (Kanto, anhydrous 95%) solution (*n*_*L*_ = 1.44), and a mixture of 40 M glycerin and 6 M CaCl_2_ (*n*_*L*_ = 1.46) to calibrate the measurement of *n*_*L*_. Mixtures of sucrose (Junsei, guaranteed reagent) and DI water with concentrations varying from 0 to 60 wt%, of which *n*_*L*_ vary linearly from 1.33 to 1.44 were used to evaluate the versatility of the proposed measurement system. A commercial hand-held refractometer (ATAGO^®^, R-5000) having a precision of 0.001 was used to check the accuracy of *n*_*L*_ measurement by the proposed system. To avoid the effect of temperature on *n*_*L*_, all experiments were conducted at 25 ± 1 °C. A white light-emitting diode and a diffuser film were used to uniformly illuminate light. To avoid the dispersion effect and illuminate monochromatic light of wavelength 589 nm, we used an orange-colored filter in front of the light source. Images of GSP were captured using a digital single-lens reflex camera (Canon, 650D) and macro lens (Canon, MP-E 65 mm). The captured GSP images were processed using the Image Processing Toolbox™ of MATLAB^®^ R2012a to analyze the brightness profile in the GSP.

## Additional Information

**How to cite this article**: Kim, W. and Kim, D. S. Gradient shadow pattern reveals refractive index of liquid. *Sci. Rep*. **6**, 28191; doi: 10.1038/srep28191 (2016).

## Supplementary Material

Supplementary Information
