# Supplementary material for: Gradient shadow pattern reveals refractive index of liquid
Source: Sci Rep. 2016 Jun 15;6:28191. doi: 10.1038/srep28191 (PMC4908432; doi:10.1038/srep28191)
Supplement: Supplementary Information [file srep28191-s1.doc]

# Supplementary Information

# Gradient shadow pattern reveals refractive index of liquid

Wonkyoung Kim and Dong Sung Kim^*^

Department of Mechanical Engineering, Pohang University of Science and Technology (POSTECH), 77 Cheongam-ro, Pohang 37673, Republic of Korea

^*^To whom all correspondence should be addressed. Electronic mail: [smkds@postech.ac.kr](mailto:smkds@postech.ac.kr)


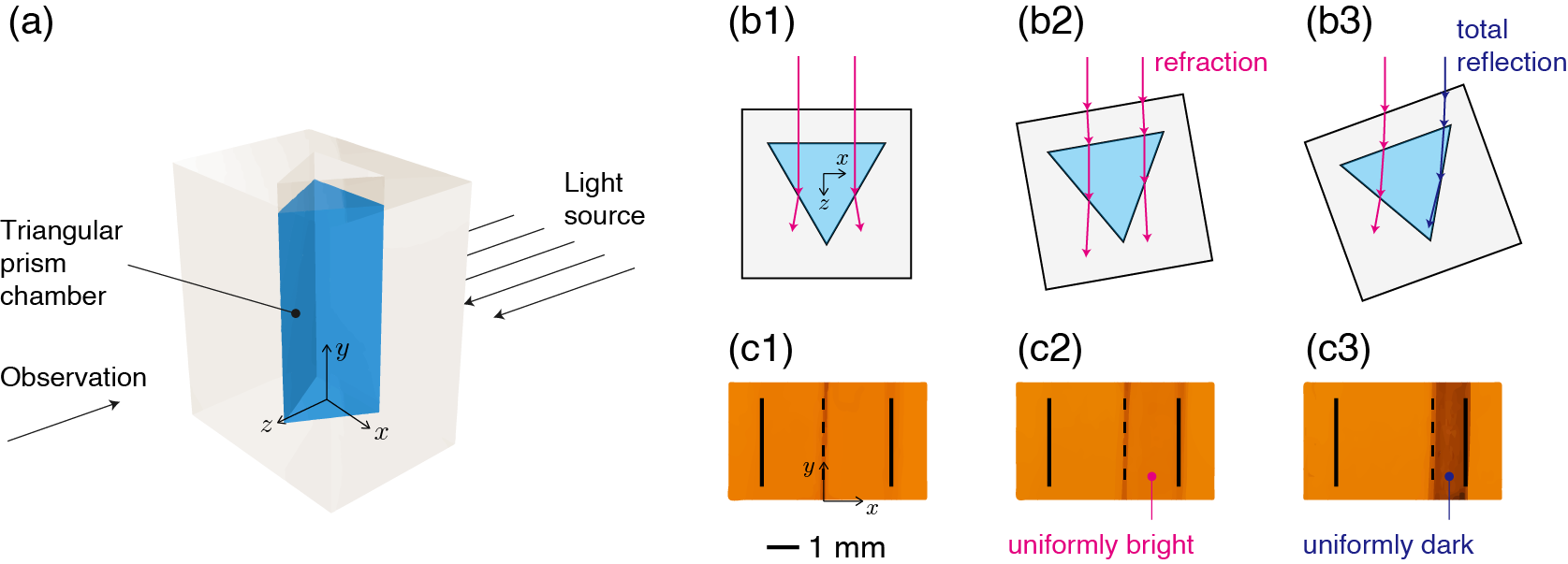


**Figure S1.** (a) Schematic diagram of the experiment using a triangular prism chamber (b) Paths of light rays passing through the triangular prism chamber with respect to rotation about the *y*-axis (c) Shadow patterns corresponding to the angles of rotation as shown in (b).


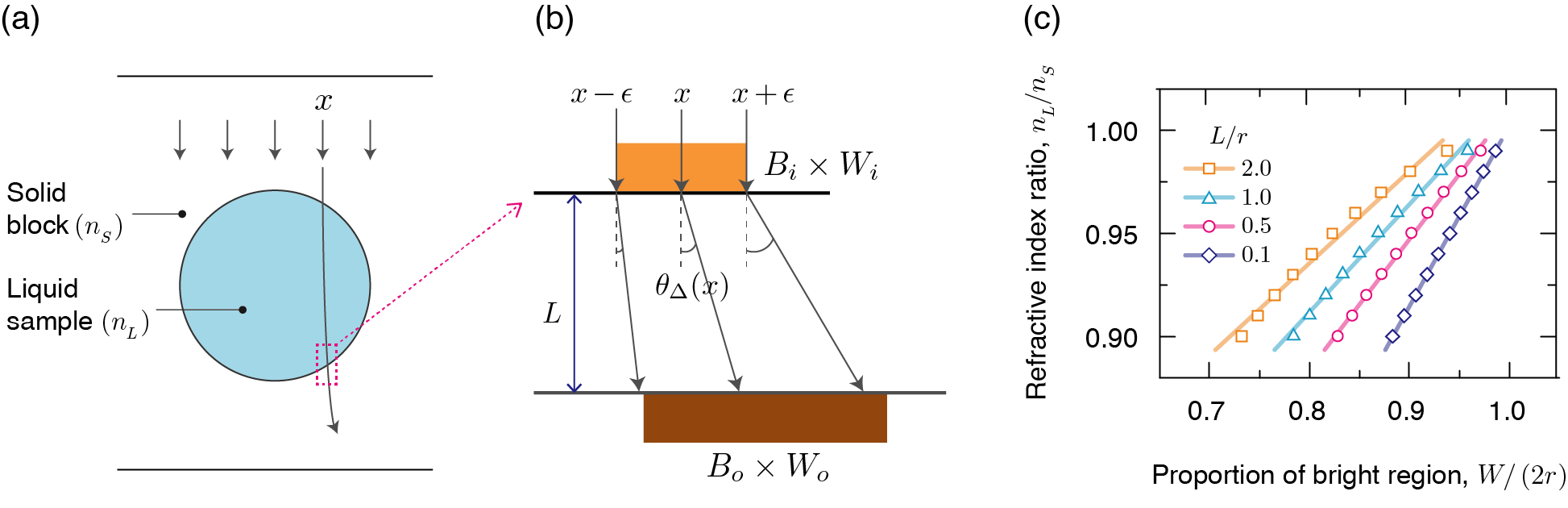


**Figure S2.** (a) Schematic diagram of paths of light rays. (b) Simplified model. (c) Relationship between the proportion of bright region in GSPs and the refractive index ratio with respect to the distance $L$ between the surface of cylindrical chamber and the location of an observation camera or a screen.

We derived a theoretical brightness profile of a GSP from the deviation angle $\theta_{\Delta}$. Passing through a cylindrical chamber, incident light spread out (Fig. S2a) and brightness (*i.e.* illumination flux) decreases. Because we dealt with 2-D problem, the inverse square law for light can be written as $B_{i}\times W_{i}=B_{o}\times W_{o}$, where $B$ and $W$ represent brightness and width of a region, respectively. Subscripts $i$ and $o$ indicate before and after passing the surface of the cylindrical chamber, respectively (Fig. S2b). Incident light coming from $\left[x-\varepsilon\right]$ to $\left[x+\varepsilon\right]$ diverges to a wider range from $\left[x-\varepsilon+L\tan\theta_{\Delta}\left(x-\varepsilon\right)\right]$ to $\left[x+\varepsilon+L\tan\theta_{\Delta}\left(x+\varepsilon\right)\right]$, where $\varepsilon$ is small length, $L$ is distance between the interface and the location of a screen. In this situation, $W_{i}$ is $2\varepsilon$ and $W_{o}$ is $2\varepsilon+L\left[\tan\theta_{\Delta}\left(x+\varepsilon\right)-\tan\theta_{\Delta}\left(x-\varepsilon\right)\right]$; the brightness profile $B_{o}$ becomes as:

$B_{0}=B_{i}\left\{1+\frac{L}{2\varepsilon}\left[\tan\theta_{\Delta}\left(x+\varepsilon\right)-\tan\theta_{\Delta}\left(x-\varepsilon\right)\right]\right\}^{-1}$. (S1)

When $\varepsilon$ approaches 0, the brightness profile is finally obtained as:

$B_{0}=B_{i}\left[1+L\frac{\text{d}}{\text{d}x}\tan\theta_{\Delta}\left(x\right)\right]^{-1}$, (S2)

which is plotted in Fig. 2c. $B_{o}$ attains its maximum value at $x=0$ as:

$B_{\max}=B_{i}\left[1+\frac{2L}{r}\left(\frac{n_{s}}{n_{L}}-1\right)\right]^{-1}$. (S3)

To find out the position of borderline between bright and dark region, we should find $W$, which is satisfying $B_{o}\left(x=\frac{W}{2}\right)=0.5B_{\max}$, that is as follows:

$B_{i}\left[1+L\left.\frac{\text{d}}{\text{d}x}\tan\theta_{\Delta}\left(x\right)\right|_{x=\frac{W}{2}}\right]^{-1}=0.5B_{i}\left[1+\frac{2L}{r}\left(\frac{n_{S}}{n_{L}}-1\right)\right]^{-1}$. (S4)

Eq. (S4) was solved numerically, and plotted in Fig. S2c.


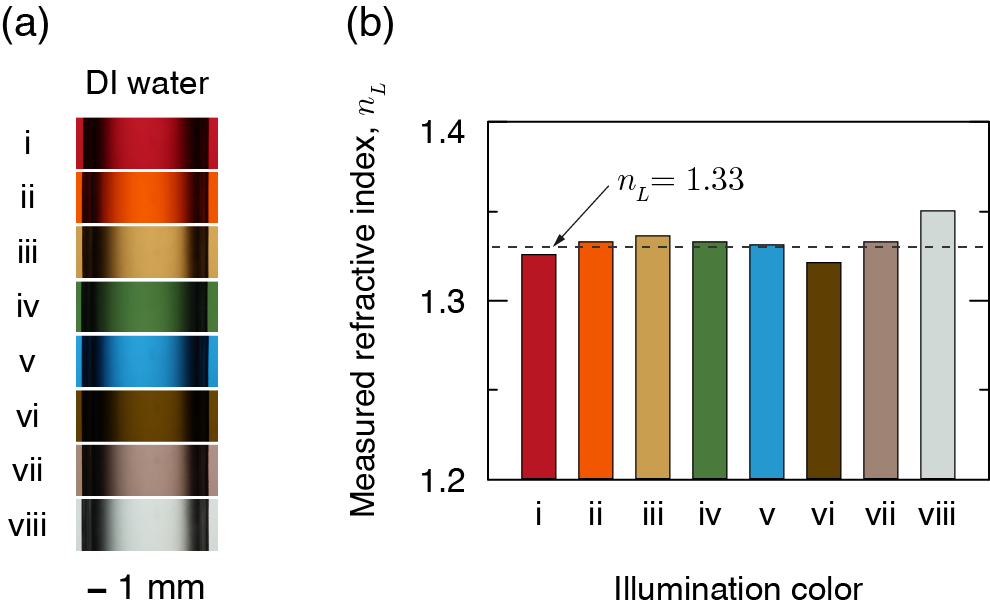


**Figure S3.** Effect of illumination color on (a) images of GSP and (b) estimates of *n_L_*. Images of GSP of DI water were taken through i-red, ii-orange, iii-yellow, iv-green, v-blue, vi-dark brown, and vii-pale brown filter, while viii-GSP image was taken without a color filter. The measured values of refractive index were found to be consistent within the error range of the proposed method (± 0.01) regardless of the color of the used filters. However, in the absence of a color filter, *n_L_* was slightly overestimated (*n_L_* = 1.35).


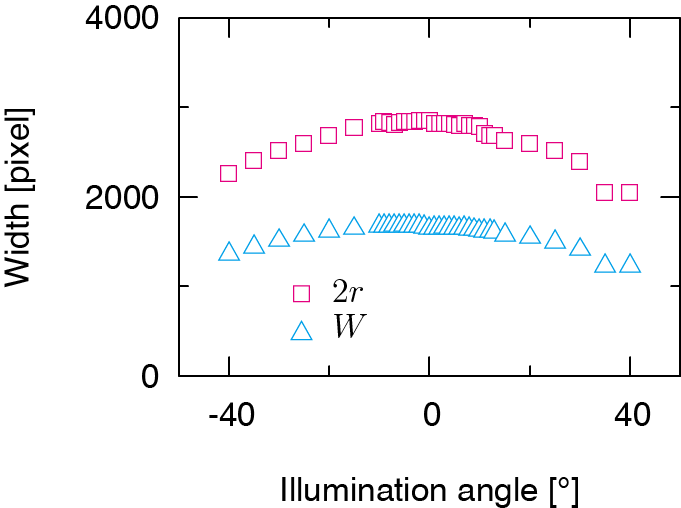


**Figure S4.** Observed widths of the cylindrical hole (red square) and the bright region (blue triangle) in the GSPs of DI water with respect to the angle of illumination direction. As the angle of illumination direction increases, both of the widths decreases.
